# Instant labeling of therapeutic cells for multimodality imaging

**DOI:** 10.7150/thno.39554

**Published:** 2020-05-15

**Authors:** Hossein Nejadnik, Kyung Oh Jung, Ashok J. Theruvath, Louise Kiru, Anna Liu, Wei Wu, Todd Sulchek, Guillem Pratx, Heike E. Daldrup-Link

**Affiliations:** 1 Department of Radiology, Molecular Imaging Program at Stanford, Stanford University, CA, 94305, USA; 2 Department of Radiation Oncology, Stanford University, CA, 94305, USA; 3 Department of Diagnostic and Interventional Radiology, University Medical Center of the Johannes Gutenberg-University Mainz, 55131 Mainz, Germany; 4 Department of Biomedical Engineering, Georgia Institute of Technology, Atlanta, GA, 30332, USA; 5 Department of Pediatrics, Stanford University, CA, 94305, USA

**Keywords:** mechanoporation, microfluidic device, iron oxide nanoparticles, ^18^F-FDG, in vivo cell tracking

## Abstract

Autologous therapeutic cells are typically harvested and transplanted in one single surgery. This makes it impossible to label them with imaging biomarkers through classical transfection techniques in a laboratory. To solve this problem, we developed a novel microfluidic device, which provides highly efficient labeling of therapeutic cells with imaging biomarkers through mechanoporation.

**Methods:** Studies were performed with a new, custom-designed microfluidic device, which contains ridges, which compress adipose tissue-derived stem cells (ADSCs) during their device passage. Cell relaxation after compression leads to cell volume exchange for convective transfer of nanoparticles and nanoparticle uptake into the cell. ADSCs were passed through the microfluidic device doped with iron oxide nanoparticles and ^18^F-fluorodeoxyglucose (FDG). The cellular nanoparticle and radiotracer uptake was evaluated with DAB-Prussian blue, fluorescent microscopy, and inductively coupled plasma spectrometry (ICP). Labeled and unlabeled ADSCs were imaged* in vitro* as well as *ex vivo* in pig knee specimen with magnetic resonance imaging (MRI) and positron emission tomography (PET). *T*_2_ relaxation times and radiotracer signal were compared between labeled and unlabeled cell transplants using Student T-test with p<0.05.

**Results:** We report significant labeling of ADSCs with iron oxide nanoparticles and ^18^F-FDG within 12+/-3 minutes. Mechanoporation of ADSCs with our microfluidic device led to significant nanoparticle (> 1 pg iron per cell) and ^18^F-FDG uptake (61 mBq/cell), with a labeling efficiency of 95%. The labeled ADSCs could be detected with MRI and PET imaging technologies: Nanoparticle labeled ADSC demonstrated significantly shorter *T*_2_ relaxation times (24.2±2.1 ms) compared to unlabeled cells (79.6±0.8 ms) on MRI (p<0.05) and ^18^F-FDG labeled ADSC showed significantly higher radiotracer uptake (614.3 ± 9.5 Bq / 1×10^4^ cells) compared to controls (0.0 ± 0.0 Bq/ 1×10^4^ cells) on gamma counting (p<0.05). After implantation of dual-labeled ADSCs into pig knee specimen, the labeled ADSCs revealed significantly shorter *T*_2_ relaxation times (41±0.6 ms) compared to unlabeled controls (90±1.8 ms) (p<0.05).

**Conclusion:** The labeling of therapeutic cells with our new microfluidic device does not require any chemical intervention, therefore it is broadly and immediately clinically applicable. Cellular labeling using mechanoporation can improve our understanding of *in vivo* biodistributions of therapeutic cells and ultimately improve long-term outcomes of therapeutic cell transplants.

## Introduction

Autologous cell therapies for repair of degenerative joint diseases typically involve harvest of therapeutic cells, such as mesenchymal stromal cells (MSC) or adipose tissue-derived stem cells (ADSCs), and transplantation in one single surgery^1^, ^2^. Labeling the therapeutic cells with imaging biomarkers enables *in vivo* cell tracking with medical imaging technologies and early detection of complications of the engraftment process, such as cell death and cell loss from the transplant site^3^-^7^. Specifically, ^18^F-fluorodeoxyglucose (FDG) labeling can be used to quantify ADSC delivery and engraftment in the target tissue with positron emission tomography (PET), and nanoparticle labeling can be used for long-term evaluations of graft retention with magnetic resonance imaging (MRI).

**Figure 1:**
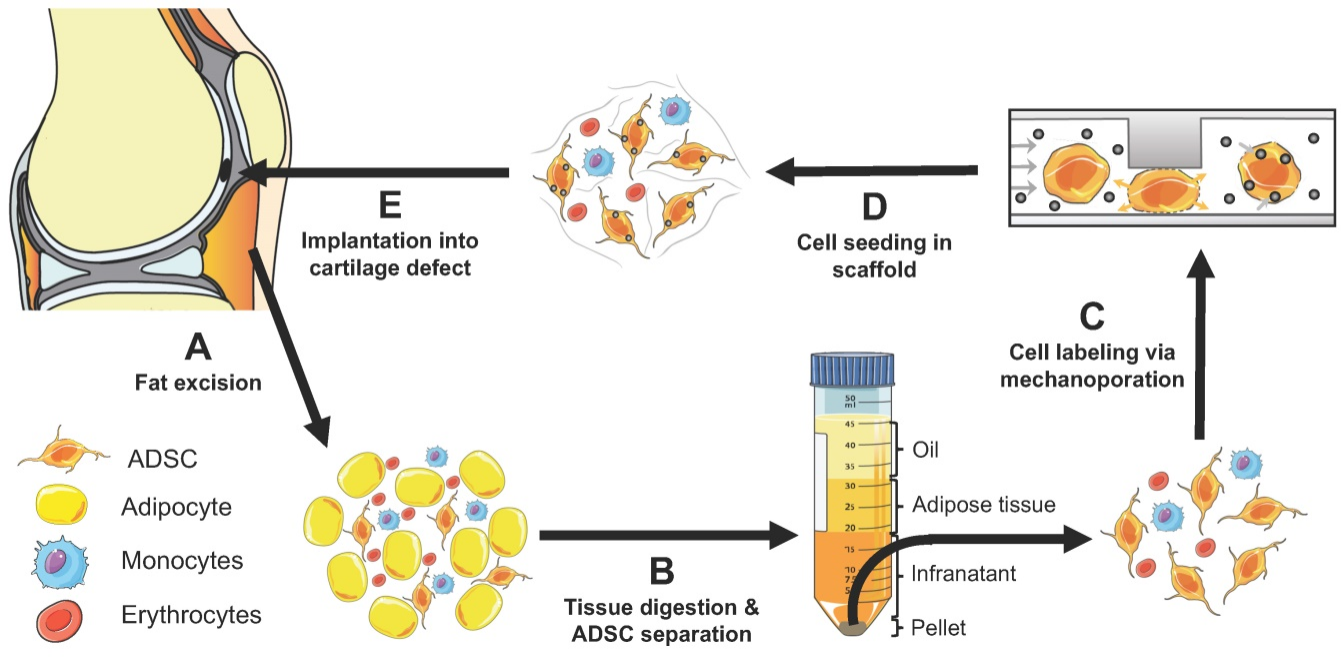
** Concept of instant ADSC harvest and labeling with imaging biomarkers.** (A) Therapeutic cells are harvested from the pre-patellar fat pad. (B) ADSCs, adipocytes and monocytes are isolated through collagenase digestion and centrifugation. (C) The harvested cells are passaged through a novel microfluidic device, which provides instant labeling through cell compression and convective transfer of imaging biomarkers. (D) The labeled cells are seeded in scaffold. (E) The labeled cells in scaffold are implanted into cartilage defects. The engraftment of the labeled cells can be tracked *in vivo* with clinical imaging technologies.

However, until now, the only ways to label ADSCs for *in vivo* imaging have required manipulation of the cells in the laboratory. Upon extraction, the cells had to be incubated with contrast agents for several hours, washed, centrifuged and then transplanted^4^, ^8^. These manipulations are problematic for clinical translation because they could lead to cell sample contamination^9^ or alterations in cell biology^10^-^12^. Most transfection agents, which are needed to shuttle imaging agents into stem cells, are not FDA-approved^13^, may induce toxic effects^14^-^17^ or alter stem cell biology^18^. In addition, ADSC labeling procedures in the laboratory take too long. In a clinical setting, ADSCs are harvested and transplanted within one surgery. To solve these problems, we developed a new technique for ADSC labeling based on simple passage of harvested cells through a novel microfluidic device, which provides instant labeling through *cell* compression and *convective transfer of imaging biomarkers.* This allows for ADSC harvest, isolation, microfluidic device passage, imaging biomarker labeling and transplantation in one session (Figure 1). Previous studies have shown that cell compression can be used for delivery of a wide range of molecules into different types of cells via diffuse delivery through transient membrane pores^19^-^21^. However, to our knowledge nobody has investigated instant stem cell labeling using cell compression and convective transfer of clinically available contrast agents. Tracking the biodistribution of therapeutic cells can improve our understanding of tissue engraftment and regeneration processes and facilitate early interventions in case of complications^22^-^25^.

The purpose of our study was to test the ability of this new microfluidic device to label therapeutic cells within 15 minutes or less such that the labeled cells can be detected with MRI and PET.

## Materials and Methods

### Microfluidic device design and production

We designed a customized microfluidic device for this project, with one inlet, five mechanoporation channels and one outlet. The mechanoporation channels contained chevron ridges with a gap size of 9.6 µm to achieve approximately 40% compression of ADSCs processed through the device. The ridges were angled to clear cell aggregates and debris that would otherwise clog the device. A pilot study demonstrated that a five-channel device successfully processed 50 million cells in 10 minutes without clogging.

The microfluidic device was molded into polydimethylsiloxane (PDMS) using a reusable SU-8 mold. Multiple devices were formed with a 10:1 ratio of PDMS and crosslinking agent that was mixed and poured onto the SU-8 mold. The PDMS was then degassed under a light vacuum chamber and cured for 1 hr at 80 °C. The PDMS was then peeled from the molds and inlets and outlets were punched using biopsy punches. The PDMS was then bonded to cleaned microscope glass slides using a plasma bonder (PDC-32G Harrick) followed by 1 hr bake at 80 °C.

### Imaging biomarkers

To achieve labeling of therapeutic cells with imaging biomarkers, we doped the microfluidic device with ferumoxytol nanoparticles and ^18^F-FDG radiotracer. Ferumoxytol (Feraheme ®, AMAG Pharmaceuticals Inc., Cambridge, MA, USA) is an iron supplement, which is FDA-approved for treatment of anemia in adult patients with renal insufficiency^26^-^30^. Due to its superparamagnetic properties, ferumoxytol can be used “off label” as a contrast agent for MRI^31^. Ferumoxytol consists of iron oxide nanoparticles, which have a mean hydrodynamic diameter of 30 nm, a Zeta potential of -24.4 ±9.32, an r1 relaxivity of 15 s^-1^mM^-1^ and an r2 relaxivity of 89 s^-1^mM^-1^ at 1.5T and 37°C, 40 Hz^32^.

In addition, we used the radiopharmaceutical ^18^F-FDG, a glucose analogue used clinically for assessment of tissue metabolism with positron emission tomography^33^. Medical-grade ^18^F-FDG was obtained from the Stanford Radiochemistry Facility.

### Adipose tissue-derived stem cells

Adipose tissue-derived stem cells were harvested from knee joints of Goettingen minipigs, using established techniques in our lab^34^. Briefly, <1mm^3^ tissue samples from the infrapatellar fat pad were collected, placed in type I collagenase (1.5mg/ml, Sigma Aldrich) for dissociation, centrifuged and then cultured in adipose-derived stem cell specific media. The cultured cells were characterized with specific surface markers for ADSCs according to the International Society for Cell Therapy (ISCT)^35^ criteria, including CD29, CD44, CD71, CD90, CD105/SH2, SH3 and the widely recognized stem cell marker STRO-1 with lack of CD31, CD45 and CD106.

### Iron oxide labeling efficiency

We and others previously showed that a cell load of at least 1 picogram Fe per cell is required for cell detection with MRI 36, 37. We also noted that that a cell load of less than 10 pg Fe per cell does not impair chondrogenesis 3, 38, 39. In a pilot study, we doped the microfluidic device with ferumoxytol at increasing concentrations of 1, 5, 10, 15 and 20 mg iron/ml, processed 1×10^7^ ADSCs through the device and determined the cellular iron uptake with inductively coupled plasma optical emission spectroscopy (ICP-OES). We found that a concentration of 10 mg iron/ml led to a cellular iron uptake between our target of 1-10 pg per cell. Therefore, further studies were done with a concentration of 10 mg iron/ml. We measured the cell processing time through the device for every experiment. We chose a flow rate of 0.5 ml/min, because it translates in a five-channel device to ~70 mm/s flow velocity in each channel. According to flow rate data from Liu et al. 70 mm/s should yield close to maximum volume change 40.

The viability of ADSCs before and after passage through the microfluidic device was tested with Trypan blue (Invitrogen) and the colorimetric Cell Counting Kit-8 (CCK-8, Sigma-Aldrich, St Louis, MO, USA), which uses a water-soluble tetrazolium salt to quantify the number of live cells by producing an orange formazan dye upon bio-reduction in the presence of an electron carrier. To evaluate the efficiency of the ferumoxytol labeling, 1×10^7^ ADSCs were labeled with 10 mg/ml FITC-Ferumoxytol and unlabeled ADSCs were used as control. The percentage of cells labeled with FITC-Ferumoxytol was quantified by flow cytometry analysis (BD FACSAria Fusion sorter, BD Bioscience, San Jose, CA, USA).

Next, we investigated whether ferumoxytol labeled ADSCs could be detected with MRI. We labeled triplicate samples of 1×10^6^ ADSCs with ferumoxytol, using the microfluidic device. Ferumoxytol-labeled and unlabeled ADSCs were transferred to test tubes and underwent MR imaging on a 3T MR scanner (Signa HDxt, GE Healthcare, Milwaukee, Wisconsin), using a flex coil (GE Healthcare, Milwaukee, Wisconsin). MRI included proton density weighted (PD) fast spin echo (FSE) with fat saturation (repetition time (TR) = 2700 ms / echo time (TE) = 32 ms / flip angle (FA) = 110º / matrix size 192 x 192 / slice thickness (SL) = 1 mm / field of view (FOV) = 14 cm / acquisition time (TA) =16 min), and multi-echo spin echo (TR=1200 / TE = 10,20,30,40,50,60,70,80/ FA = 90/ matrix size 192 x 192/ SL = 1.1/ FOV = 14/ TA = 13 min) sequences.

To analyze the MRI data, we generated *T*_2_-relaxation time maps using Osirix software (Pixmeo SARL, Bernex, Switzerland) and measured *T*_2_-relaxation times of each implant by operator-defined regions of interest 3.

To compare mechanoporation and conventional ferumoxytol labeling, triplicates of 1×10^6^ ADSCs were co-incubated with FITC-conjugated ferumoxytol or ferumoxytol alone (10 mg Fe/ml) for 12 minutes. Cells were washed twice with phosphate buffered saline (PBS) to remove residual ferumoxytol and then stained for cell nucleus (DAPI), cytoskeleton (Phalloidin) and FITC-conjugated ferumoxytol. In addition, DAB-enhanced Prussian blue staining was performed.

### Cell radiolabeling efficiency

^18^F-FDG labeling provides a clinically translational approach for PET/MRI tracking of ADSCs. The efficiency of the labeling procedure was investigated for two approaches, microfluidics-based mechanoporation and conventional passive co-incubation. A single-cell suspension of ADSCs (5×10^6^) was mixed with medical-grade ^18^F-FDG (57 MBq/ml; 1 ml ^18^F-FDG [344 MBq] mixed with 2 ml FACS buffer [3x] and 3 ml PBS) and passed once through the microfluidic device (5 channels, flow rate 0.5 ml/min). We measured the cell processing time through the device for every experiment. Another batch of ADSCs (1×10^6^) was incubated with ^18^F-FDG (30 MBq/ml; 0.1 ml ^18^F-FDG [33 MBq] mixed with 1 ml glucose-free DMEM) for 12 min at 37°C. The labeled cells were washed twice with PBS to remove residual radioactivity and 5×10^4^ cells were transferred to a 24-well plate for PET imaging (Inveon D-PET, Siemens Preclinical Solutions, Knoxville, TN) using an acquisition time of 10 min and an energy window of 425-650 keV. Unlabeled ADSCs (5×10^4^) were used as control. After PET acquisition, the radioactivity per well was quantified by region of interest (ROI) analysis using the Inveon Research Workplace (IRW) software. Another set of 1×10^4^ labeled cells was measured using an automatic gamma counter (Hidex, Turku, Finland).

### Dual-modality imaging of therapeutic cells in pig knee joints

To evaluate, if the microfluidic device assisted mechanoporation procedure enables dual-modality imaging of therapeutic cells in pig knee joints, we doped the microfluidic device with 10 mg/ml Ferumoxytol and 74 MBq/ml ^18^F-FDG and processed quadruple samples of 1×10^7^ ADSCs through the device. An additional quadruple set of 1×10^7^ ADSCs was processed through a microfluidic device doped with PBS. The dual labeled (n=4) and unlabeled (n=4) cells were implanted into eight full-thickness cartilage defects in the distal femur of pig knee specimen. The therapeutic cell transplants were secured with fibrin glue (Evicel®, Ethicon, Somerville, NJ) (Figure 1D and E) and the joint capsule, muscles and skin were closed with sutures.

Pig knees underwent simultaneous PET/MRI after stem cell implantation, using a clinical 3T Signa PET/MR scanner (GE Healthcare, Milwaukee, Wisconsin) equipped with a flex coil (GE Healthcare, Milwaukee, Wisconsin). MRI included proton density weighted (PD) fast spin echo (FSE) with fat saturation (repetition time (TR) = 2700 ms / echo time (TE) = 32 ms / flip angle (FA) = 110º / matrix size 192 x 192 / slice thickness (SL) = 1 mm / field of view (FOV) = 14 cm / acquisition time (TA) =16 min), and multi-echo spin echo (1200/ 10,20,30,40,50,60,70,80/ 90/ 192 x 192/ 1.1/ 14/ 13) sequences. PET images were obtained with an acquisition time of 30 min and 3D time-of-flight ordered-subsets expectation-maximization reconstruction.

To analyze the MRI data, we generated *T*_2_-relaxation time maps and measured *T*_2_-relaxation times of each implant as described earlier. To analyze the PET dataset, we used the scanner specific software (Image QC, GE Healthcare, Milwaukee, Wisconsin) and measured MBq/ml for each implant by operator-defined regions of interest.

### Statistical analysis

All experiments were performed with cells from at least three donors and repeated at least three times. All quantitative parameters were compared between different experimental groups, using an unpaired student *t*-test and considering a *p*<0.05 to indicate statistical significance. Statistical analysis was performed using GraphPad Prism (version 6.0).

## Results

### Instant labeling of ADSCs with ferumoxytol nanoparticles with a novel microfluidic device

**Figure 2:**
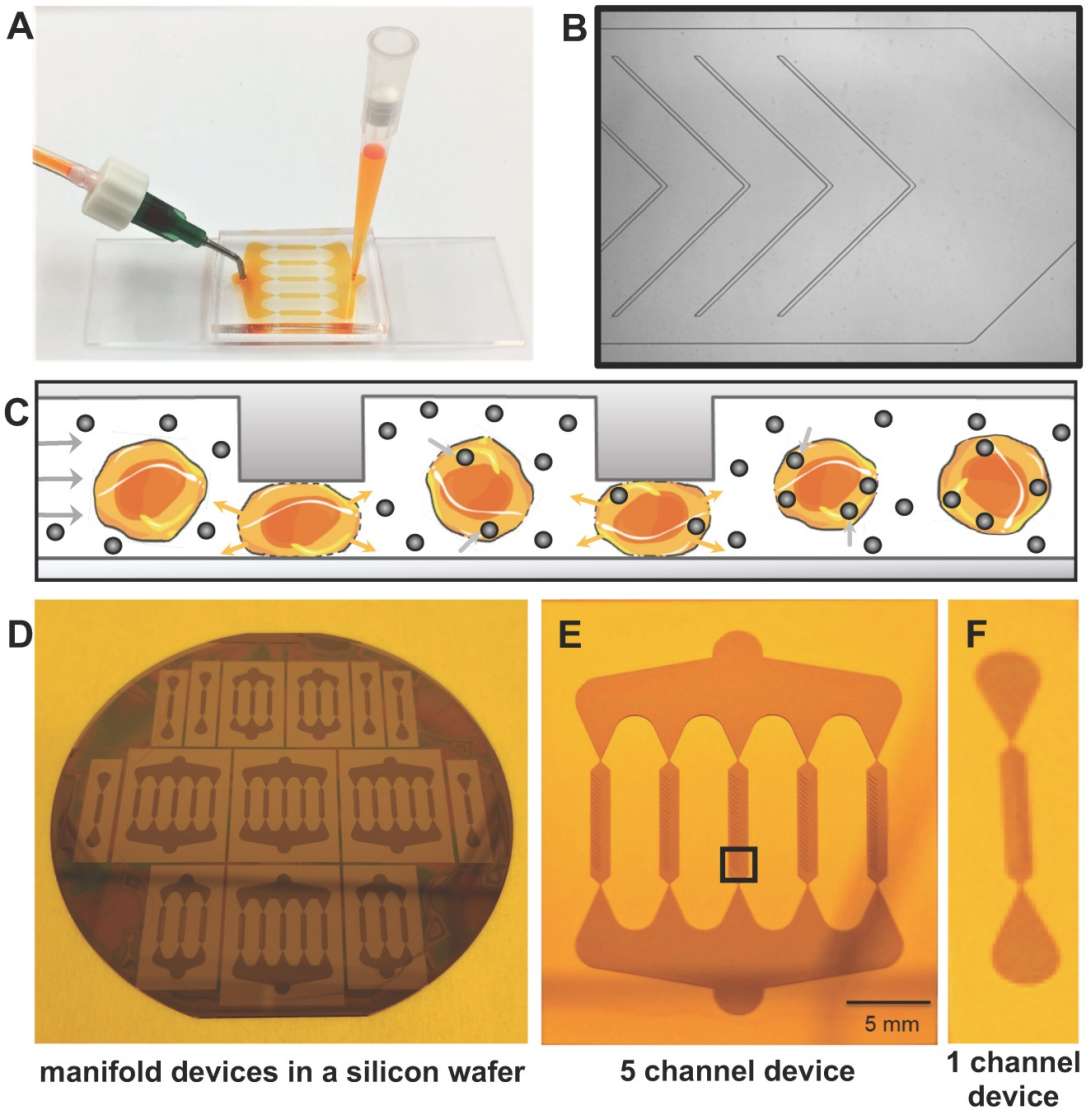
** Microfluidic device for instant cell labeling with imaging biomarkers. (**A) Setup of a prototype microfluidic device with one inlet and one outlet, (B) Phase contrast image (corresponds to black box in E) of one channel of the microfluidic device with multiple ridges (C) Schematic cross-sectional view of therapeutic cells undergoing repeated compression under the ridges and expansion/relaxation after passing the ridges. The relaxation state leads to a transient increase in cell membrane permeability with convective uptake of nanoparticles and radiotracers. (D) Image of the silicon wafer used to fabricate the microfluidic devices. Image of (E) the 5-channel and (F) 1-channel microfluidic device mold for large and small cell samples.

**Figure 3:**
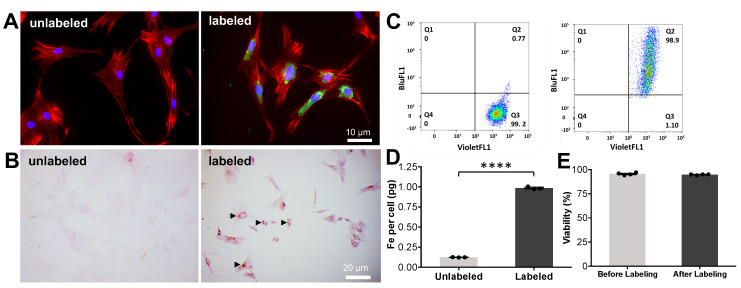
** Instant labeling of ADSCs with ferumoxytol nanoparticles using the novel microfluidic device.** (A) Fluorescence microscopy of ADSCs before (unlabeled) and after (labeled) processing through the microfluidic device doped with FITC-conjugated ferumoxytol (10 mg Fe/ml). Labeled ADSCs demonstrate green fluorescent signal in the cytoplasm, confirming accumulation of ferumoxytol nanoparticle uptake (blue represents DAPI, red represents a phalloidin probe which selectively stains F-actin cytoskeleton and green represents FITC-conjugated ferumoxytol). (B) DAB-enhanced Prussian blue staining shows brown precipitation (arrow heads) in the cytoplasm of ADSCs, consistent with cellular accumulation of ferumoxytol nanoparticles. (C) Flow cytometry analysis of ADSCs demonstrates more than 95% labeling efficiency. (D) Iron content of ferumoxytol labeled cells, as determined by inductively coupled plasma optical emission spectroscopy shows significantly higher cellular iron uptake of ferumoxytol labeled cells compared to unlabeled controls (p < 0.0001). (E) Trypan blue assay demonstrated no significant difference in cell viability of ferumoxytol labeled cells compared to unlabeled controls. All quantitative data represent mean data ± SEM of three cell samples per experimental group; *p < 0.05;* unpaired *t* test.

Our prototype microfluidic device is shown in Figure 2. The device is composed of one inlet, one outlet and one or more mechanoporation channels, depending on the number of therapeutic cells and volume of the cell suspension to be processed. Each mechanoporation channel contains multiple ridges with a vertical gap size that is tailored to the size of the specific therapeutic cell type with the goal to achieve a cell compression by approximately 40%. Therefore, we created ridges with a gap size of 9.6 µm for ADSCs, which have a diameter of 13-17 µm. ADSCs flow through the mechanoporation channel and pass under the ridges where they undergo rapid compression and lose some intracellular volume. After each ridge compression and volume loss event, the cells recover in shape and volume, causing the cell to uptake surrounding fluid and molecules (Figure 2C). The cell volume loss and recovery behavior leads to a transient increase in cell membrane permeability and intracellular uptake of target molecules by convective fluid transport 40. The mean nanoparticle labeling time was 12 ± 3 minutes. We confirmed cellular uptake of FITC-labeled ferumoxytol by ADSCs with fluorescence microscopy (Figure 3A) and DAB-enhanced Prussian blue staining (Figure 3B).

Fluorescence activated cell sorting showed greater than 95% ADSC labeling efficiency (Figure 3C). ADSCs processed through the microfluidic device doped with ferumoxytol (10 mg Fe/ml) showed significant nanoparticle uptake (1.07 ± 0.05 pg per cell) compared to controls (0.12 ± 0.002 pg per cell), as determined by inductively coupled plasma optical emission spectroscopy (ICP-OES) (Figure 3D). The viability of microfluidic device processed and unprocessed ADSCs, as measured by Trypan blue staining, was not significantly different (Figure 3E). Transmission electron microscopy (TEM) showed iron oxide nanoparticles in secondary lysosomes (Figure S1). By comparison, conventional co-incubation of ADSCs with FITC-conjugated ferumoxytol or ferumoxytol alone (10 mg Fe/ml) for 12 minutes did not show detectable uptake into ADSCs by fluorescent imaging or DAB-enhanced Prussian blue staining, respectively (Figure S2).

Further *in vitro* validation studies revealed that dual labeling had no effect on the migration (Figure S4), proliferation (Figure S5), immunomodulation (Figure S6) and differentiation (Figure S7) of ADSCs. The labelling efficiency of iron oxide nanoparticles with different physiochemical properties (ferumoxytol, ferucarbotran, molday ION evergreen) was compared. Uptake of the positively charged nanoparticle; molday ION evergreen was higher in ADSCs compared to uptake of the negatively charged iron oxide nanoparticles (Figure S8). Due to our interest in developing a stem cell labeling approach that is translatable, ferumoxytol was used in subsequent experiments because it is an FDA approved nanoparticle.

### Microfluidic device assisted cell labeling enables cell detection with MRI and PET

Imaging can non-invasively and quantitatively assess the distribution of stem cells following autologous transfer. Next, we demonstrated that microfluidics-based mechanoporation can be used to label stem cells simultaneously with ferumoxytol nanoparticles and ^18^F-FDG for sequential or combined MRI and PET imaging. While many biomarkers are available to label stem cells, ferumoxytol nanoparticles and ^18^F-FDG were selected for this project in anticipation of future clinical translation. Ferumoxytol is FDA approved as an iron supplement and can be used “off label” as a contrast agent for MRI. ^18^F-FDG is the standard radiotracer for clinical PET imaging, and its use for labeling stem cells is well documented 41, 42. However, conventional cell labeling relies on active transport of ferumoxytol nanoparticles or ^18^F-FDG by cells and thus requires long incubation times (30-60 min). Here, we investigate microfluidic device assisted mechanoporation as a new approach for instant intraoperative labeling of autologous stem cells.

**Figure 4:**
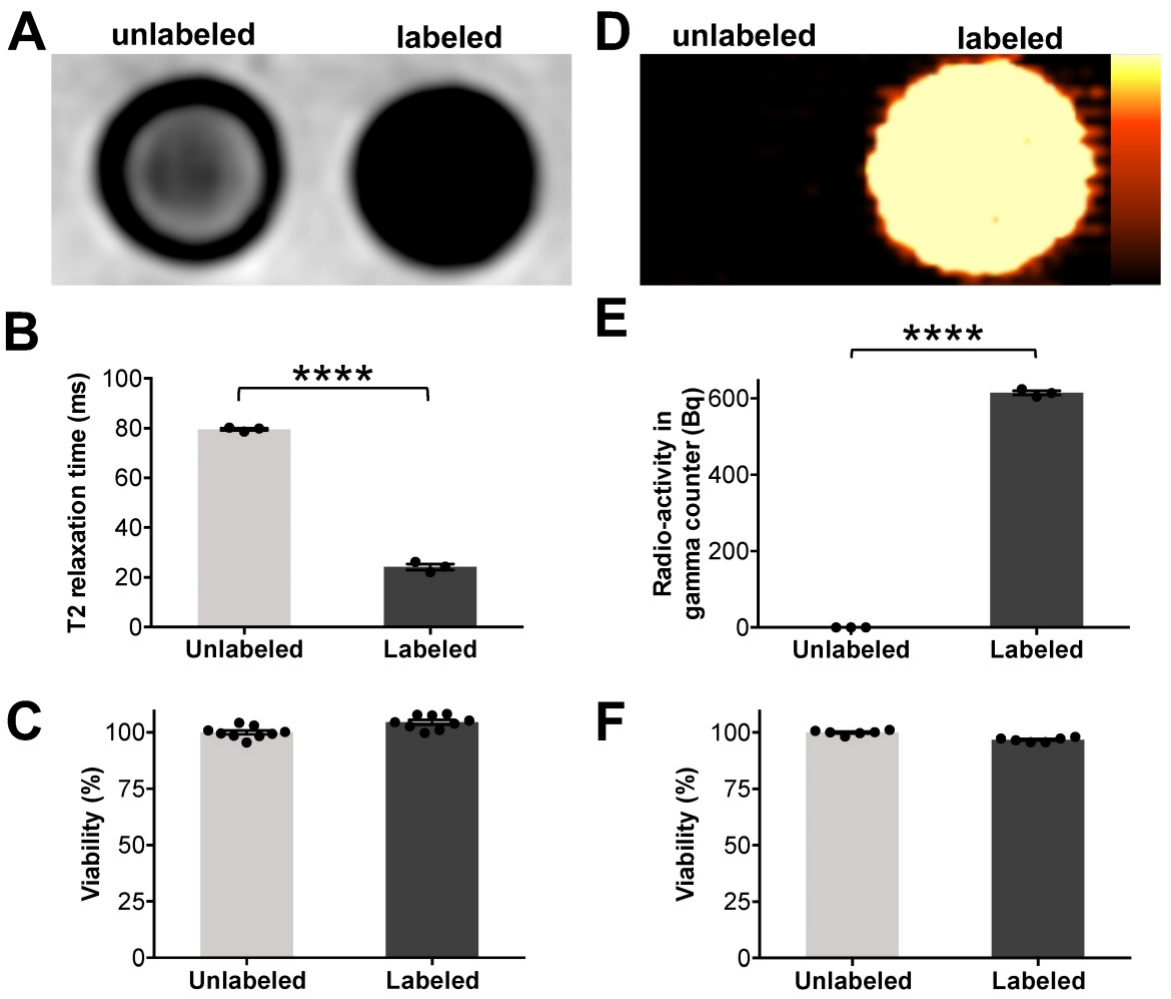
** Microfluidic device assisted cell labeling enables cell detection with PET and MR imaging.** ADSCs were labeled with ferumoxytol nanoparticles and a separate set of cells was labeled with ^18^F-FDG, using microfluidic device-assisted mechanoporation. The cells were transferred to test tubes and scanned with clinical MRI and PET: (A) MRI shows marked hypointense (dark) *T*_2_-signal of 1×10^6^ ferumoxytol labeled ADSCs compared to unlabeled cells. (B) Corresponding quantitative data confirmed significant shortening of *T*_2_ relaxation times of labeled ADSCs compared to unlabeled ADSCs (p < 0.0001). (C) Viability of ferumoxytol-labeled and unlabeled cells, measured with a Cell Counting Kit-8 (CCK-8) assay was not significantly different. (D) PET imaging demonstrates high PET signal of ^18^F-FDG-labeled ADSCs (5×10^4^) compared to unlabeled cells. (E) ^18^F-FDG uptake quantification by gamma counting confirms significantly higher radioactivity in ^18^F-FDG-labeled ADSCs compared to unlabeled ADSCs (p < 0.0001). (F) No significant difference in viability is observed between ^18^F-FDG-labeled and unlabeled cells. All data are shown as mean ± SEM of three cell samples per experimental group; *p* < 0.05; unpaired *t* test.

We doped the microfluidic device with ferumoxytol (10 mg Fe/ml) and ^18^F-FDG (57 MBq/ml) and processed a suspension of ADSCs (5×10^6^ cells/ml) through the device. Triplicate samples of 1×10^6^ processed cells and unprocessed controls were transferred to Eppendorf test tubes and underwent MR imaging on a 3T clinical MR scanner (Signa HDxt, GE Healthcare, Milwaukee, Wisconsin). *T*_2_-weighted fast spin echo sequences showed a marked hypointense (dark) signal enhancement of ferumoxytol-labeled cells compared to unlabeled controls (Figure 4A).

These findings were quantitatively confirmed by significantly shorter *T*_2_ values of ferumoxytol-labeled ADSCs (24.2 ± 2.1 ms / 1×10^6^ cells) compared to unlabeled ADSCs (79.6 ± 0.8 ms / 1×10^6^ cells) (Figure 4B). A colorimetric cell viability assay found no significant difference in viability of ferumoxytol labeled and unlabeled cells (Figure 4C). Another set of labeled cells were dispensed in 24-well plates (5×10^4^ cells/well) and imaged using a preclinical PET scanner (Inveon D-PET, Siemens, Knoxville, TN). ^18^F-FDG-labeled ADSCs demonstrated strong radiotracer signal on PET images while unlabeled ADSCs showed no detectable radiotracer signal (Figure 4D). These findings were quantitatively confirmed by gamma counting (Figure 4E), which revealed that ^18^F-FDG-labeled ADSCs had significantly higher radioactivity (614.3 ± 9.5 Bq / 1×10^4^ cells) than unlabeled ADSCs (0.0 ± 0.0 Bq/ 1×10^4^ cells). Cell viability was not significantly affected by the radiolabeling procedure (Figure 4F).

Mechanoporation-based radiolabeling was compared with conventional cell labeling (passive co-incubation). Most cells, including stem cells, express glucose transporters (GLUT) on their surface to satisfy their glucose requirements. ^18^F-FDG is a known substrate for several GLUTs and it is actively imported by most cell types. However, GLUT expression may vary from sample to sample, leading to inconsistent labeling. Furthermore, GLUT-assisted labeling is too slow for intraoperative cell labeling. To compare microfluidic-device assisted mechanoporation and conventional ^18^F-FDG labeling, triplicate samples of 1x10^6^ ADSCs were co-incubated with ^18^F-FDG and 5x10^6^ ADSCs were processed through the microfluidics device. PET imaging revealed that cells labeled by mechanoporation had 63% higher uptake than cells labeled through conventional co-incubation (Figure S3A and S3B). These results were confirmed by gamma counting, which found that mechanoporation led to significantly higher ^18^F-FDG labeling of ADSCs (614.3 ± 9.5 Bq / 1×10^4^ cells) compared with conventional co-incubation (376.6 ± 4.5 Bq / 1×10^4^ cells; Figure S3C). There was no significant difference in cell viability between the two methods (Figure S3D).

### Microfluidic device assisted mechanoporation enables dual-modality imaging of therapeutic cells in pig knee joints

**Figure 5:**
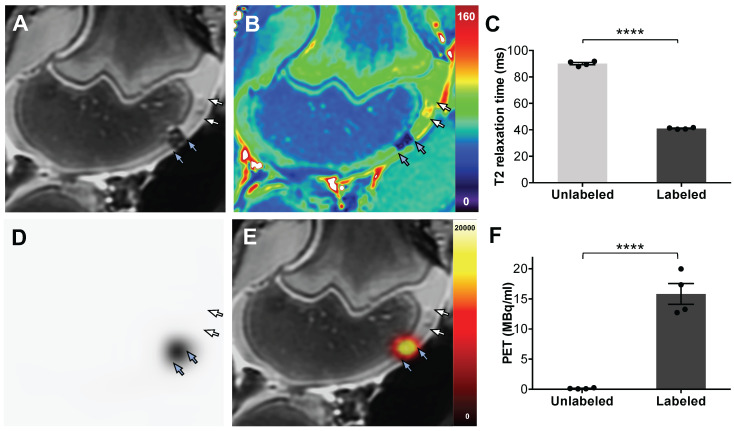
** PET/MR imaging of dual labeled ADSCs in cartilage defects of pig knees.** ADSCs were labeled with ferumoxytol nanoparticles and ^18^F-FDG, using microfluidic device-assisted mechanoporation, and implanted into cartilage defects of the distal femur of pig knees. (A) MRI enables detection of a representative nanoparticle labeled ADSC implant based on a marked hypointense (dark) signal effect on a *T*_2_-weighted scan (blue arrows). By comparison, unlabeled ADSCs are difficult to delineate from adjacent cartilage (white arrows). (B) *T*_2_ map demonstrates *T*_2_ relaxation times on a color scale, with marked *T*_2_ relaxation time shortening of the ferumoxytol labeled implant (blue arrows) compared to the unlabeled implant (white arrows). (C) Corresponding quantitative data confirmed significant shortening of *T*_2_ relaxation times of labeled implants compared to unlabeled implants (p < 0.0001). (D) PET image of the same knee joint as in (A) shows marked PET signal of the dual labeled ADSC implant (blue arrows), while the unlabeled implant shows no detectable PET signal (white arrows). (E) Fused PET/MR images show colocalization of the MRI and PET signals in the labeled implant (blue arrows). (F) Quantification of the PET signal confirms significantly higher radioactivity (15.8 MBq/ml) of ^18^F-FDG labeled implants compared to unlabeled implants (0.1 MBq/ml; p < 0.0001). All data are shown as mean ± SEM of four implants per group; *p* < 0.05; unpaired *t* test.

To ensure that the labeled cells can be detected in cartilage defects, an additional set of 1×10^7^ dual-labeled ADSCs (with ferumoxytol and ^18^F-FDG) were implanted into artificially created cartilage defects of pig knee joint specimen, using previously established techniques 5, 8. All specimens underwent integrated PET/MRI on a clinical PET/MRI scanner (Signa, GE Healthcare, Milwaukee, Wisconsin), using sagittal *T*_2_-weighted sequences and simultaneous PET data acquisition (Figure 5).

Dual-labeled ADSCs in cartilage defects of the distal femur showed a marked hypointense (negative) MRI signal, which enabled delineation of the therapeutic cell transplant. By comparison, unlabeled ADSCs could not be delineated from adjacent cartilage (Figure 5A). *T*_2_ relaxation time maps confirmed nanoparticle-induced shortening of *T*_2_-relaxation times in labeled ADSCs, with minimal *T*_2_ shortening of unlabeled ADSCs, (Figure 5B). Corresponding quantitative *T*_2_ relaxation times of labeled ADSC implants (41 ± 0.6 ms) were significantly decreased compared with unlabeled controls (90 ± 1.8 ms, p= < 0.0001, Figure 5C). The same dual labeled cells demonstrated marked PET signal, while unlabeled ADSCs were not detectable with PET imaging (Figure 5D). Integrated PET/MR imaging added anatomical background information and enabled simultaneous detection of the nanoparticle and ^18^F-FDG signal of dual labeled cells (Figure 5E). Quantification of the PET signal confirmed significantly higher radioactivity in labeled implants (15.8 ± 3.5 MBq/ml) compared to unlabeled implants (0.1 ± 0.08 MBq/ml) (Figure 5F).

## Discussion

Our results demonstrate that ADSCs can be labeled efficiently with a novel microfluidics device, which provides imaging biomarker uptake through mechanoporation. Previous studies have reported the use of mechanoporation for intracellular delivery of macromolecules for cell engineering applications^43^. However, to the best of our knowledge, microfluidics-assisted mechanoporation has not been explored for stem cell labeling and imaging applications. Our approach could be broadly applied for instant labeling of therapeutic cells with imaging biomarkers. This could facilitate in vivo cell tracking studies, improve our understanding of tissue regeneration processes, enable us to optimize new cell therapies and thereby, improve outcomes.

Previous approaches of mechanoporation for intracellular delivery of macromolecules included fluid shear loading 44, scrape loading 45, cavitation induction 46-48, microinjection 49, and nanoneedles 50, 51. Common limitations of these techniques were low efficiency, impaired cell viability and poor throughput. Hallow et al. used fluid shear-forces in a microchannel system to load prostate cancer cells with macromolecules. However, labeling efficiency was approximately 30% and cell viability after labeling was 80% 44. Matsumoto et al. used a nanoneedle array to deliver FITC-dextran into human embryonic kidney 293 cells and achieved a delivery efficiency of 45 ± 9% and for a relatively low cell quantity of ~10^5^ cells 50. Shalek et al. used vertical nanowires to improve the delivery of biomolecules into primary mammalian cells. However, loading cells with biomolecules took up to one hour and some cell functions were impaired by the nanowires 51. In our system, we labeled 50 million cells in a five-channel device with two different biomarkers within 15 minutes and achieved a labeling efficiency of 95% and less than 5% cell death. Our results are in line with a study by Liu et al. who reported that they were able to process 50 million cells within 10 minutes using a five-channel device 43. While most of the results presented in their study were generated using single-channel devices, our study used multi-channel devices which increase throughput on a single chip by using multiple microchannels in parallel. In addition, we used microfluidic devices, tailored to ADSCs with a gap size of 9.6µm. The devices used in our study have been specifically designed and improved for rapid processing of stem cells for clinical translation.

Electroporation is an alternative approach which utilizes electric currents to transiently increase cell membrane permeabilities and thereby, deliver macromolecules to cells with high efficiency 52-54. However, electroporation techniques impair cell viability and consequently, lead to cell loss. This is complicated in a clinical setting as it would require separation of viable and dead cells before their transplantation.

Establishing a balance between identifying a cellular iron load that is high enough to facilitate the detection of the iron labeled cells with MRI and low enough to prevent any effect on stem cell differentiation into chondrocytes is essential 3. The orientation of the chevron ridges in our mechanoporation channels can be adjusted, therefore we chose the orientation which resulted in significant MRI signal with minimum iron load, because excessive iron load can impair the chondrogenic differentiation of MSC 14. Additionally, our studies and others have demonstrated that limited quantities of iron oxide in the cells will be incorporated in regular iron metabolism resulting in unimpaired chondrogenic differentiation of the labeled stem cells 3, 4, 55-57.

Our approach has the distinct advantage that it provides highly efficient biomarker labeling of therapeutic cells with minimal or absent effects on cell viability, migration, proliferation, differentiation and immunomodulation abilities, thereby enabling “one stop” cell harvest, labeling and transplantation procedures. Our method provides efficient and fast labeling of cells, compatible with the typical timeframe of a surgical procedure. Our study did not investigate the *in vivo* or *ex vivo* biodistribution of dual-labeled ADSCs, which is of high interest for longitudinal assessment of viability and activity of stem cells after labeling and transplantation. However, our future studies will investigate *in vivo* stem cell tracking in a large animal model and also evaluate tumor trafficking of chimeric antigen receptor (CAR) T-cells in a small animal model, using microfluidic-assisted labeling. Results from these future studies will provide more insight about *in vivo* viability and activity after labeling and will provide radiological-pathological correlations. Once validated, the new microfluidic device-based cell labeling approach could be immediately applied to study the engraftment of ADSC transplants in arthritic joints of patients in ongoing clinical trials. Once established, this microfluidics device could be also applied to label other therapeutic cell types, such as chondrocytes, MSC and T-cells, among others. Therefore, our results might have major and broad health care impact, can be readily translated to the clinic, provide a novel tool to monitor stem cell engraftment and ultimately, help improve long-term outcomes. Thus, we expect our new microfluidics device to spur on tissue regeneration research and stem cell imaging applications beyond our own research focus.

In summary, we reported a novel approach for rapid and efficient labeling of stem cells with a novel microfluidics device that enables imaging biomarker labeling and detection of transplanted cells with medical imaging technologies.

## Abbreviations

ADSCs: adipose tissue-derived stem cells
FDG: ^18^F-fluorodeoxyglucose
GLUT: glucose transporters
ICP-OES: inductively coupled plasma optical emission spectroscopy
IRW: Inveon Research Workplace
ISCT: International Society for Cell Therapy
MRI: magnetic resonance imaging
MSC: mesenchymal stromal cells
PET: positron emission tomography
PDMS: polydimethylsiloxane
ROI: region of interest
TEM: Transmission electron microscopy

## References

[1] Squillaro T, Peluso G, Galderisi U (2016). Clinical Trials With Mesenchymal Stem Cells: An Update. Cell Transplant.

[2] Coughlin RP, Oldweiler A, Mickelson DT, Moorman CT 3rd (2017). Adipose-Derived Stem Cell Transplant Technique for Degenerative Joint Disease. Arthrosc Tech.

[3] Khurana A, Chapelin F, Beck G, Lenkov OD, Donig J, Nejadnik H (2013). Iron administration before stem cell harvest enables MR imaging tracking after transplantation. Radiology.

[4] Henning TD, Gawande R, Khurana A, Tavri S, Mandrussow L, Golovko D (2011). Magnetic Resonance Imaging of Ferumoxide-Labeled Mesenchymal Stem Cells in Cartilage Defects: In Vitro and In Vivo Investigations. Molecular imaging.

[5] Theruvath AJ, Nejadnik H, Lenkov O, Yerneni K, Li K, Kuntz L Tracking Stem Cell Implants in Cartilage Defects of Minipigs by Using Ferumoxytol-enhanced MRI. Radiology. 2019: 182176.

[6] Li L, Jiang W, Luo K, Song H, Lan F, Wu Y (2013). Superparamagnetic iron oxide nanoparticles as MRI contrast agents for non-invasive stem cell labeling and tracking. Theranostics.

[7] Mertens ME, Frese J, Bolukbas DA, Hrdlicka L, Golombek S, Koch S (2014). FMN-coated fluorescent USPIO for cell labeling and non-invasive MR imaging in tissue engineering. Theranostics.

[8] Nejadnik H, Taghavi-Garmestani SM, Madsen SJ, Li K, Zanganeh S, Yang P (2018). The Protein Corona around Nanoparticles Facilitates Stem Cell Labeling for Clinical MR Imaging. Radiology.

[9] FDA Inspection of Human Cells, Tissues, and Cellular and Tissue-Based Products (HCT/Ps). 2012.

[10] Kuroda T, Yasuda S, Sato Y (2013). Tumorigenicity studies for human pluripotent stem cell-derived products. Biol Pharm Bull.

[11] Motaln H, Schichor C, Lah TT (2010). Human mesenchymal stem cells and their use in cell-based therapies. Cancer.

[12] Soenen SJ, De Cuyper M (2009). Assessing cytotoxicity of (iron oxide-based) nanoparticles: an overview of different methods exemplified with cationic magnetoliposomes. Contrast Media Mol Imaging.

[13] Arbab AS, Yocum GT, Wilson LB, Parwana A, Jordan EK, Kalish H (2004). Comparison of transfection agents in forming complexes with ferumoxides, cell labeling efficiency, and cellular viability. Molecular imaging.

[14] Kostura L, Kraitchman DL, Mackay AM, Pittenger MF, Bulte JW (2004). Feridex labeling of mesenchymal stem cells inhibits chondrogenesis but not adipogenesis or osteogenesis. NMR Biomed.

[15] Sykova E, Jendelova P (2007). In vivo tracking of stem cells in brain and spinal cord injury. Prog Brain Res.

[16] Mailander V, Lorenz MR, Holzapfel V, Musyanovych A, Fuchs K, Wiesneth M (2008). Carboxylated superparamagnetic iron oxide particles label cells intracellularly without transfection agents. Mol Imaging Biol.

[17] Wilhelm C, Gazeau F (2008). Universal cell labelling with anionic magnetic nanoparticles. Biomaterials.

[18] Schafer R, Kehlbach R, Wiskirchen J, Bantleon R, Pintaske J, Brehm BR (2007). Transferrin receptor upregulation: in vitro labeling of rat mesenchymal stem cells with superparamagnetic iron oxide. Radiology.

[19] DiTommaso T, Cole JM, Cassereau L, Bugge JA, Hanson JLS, Bridgen DT (2018). Cell engineering with microfluidic squeezing preserves functionality of primary immune cells in vivo. Proc Natl Acad Sci U S A.

[20] Sharei A, Zoldan J, Adamo A, Sim WY, Cho N, Jackson E (2013). A vector-free microfluidic platform for intracellular delivery. Proc Natl Acad Sci U S A.

[21] Kollmannsperger A, Sharei A, Raulf A, Heilemann M, Langer R, Jensen KF (2016). Live-cell protein labelling with nanometre precision by cell squeezing. Nat Commun.

[22] Cao F, Lin S, Xie X, Ray P, Patel M, Zhang X (2006). In vivo visualization of embryonic stem cell survival, proliferation, and migration after cardiac delivery. Circulation.

[23] Ahrens ET, Flores R, Xu H, Morel PA (2005). In vivo imaging platform for tracking immunotherapeutic cells. Nat Biotechnol.

[24] Wu TJ, Tzeng YK, Chang WW, Cheng CA, Kuo Y, Chien CH (2013). Tracking the engraftment and regenerative capabilities of transplanted lung stem cells using fluorescent nanodiamonds. Nat Nanotechnol.

[25] Chen Z, Yan C, Yan S, Liu Q, Hou M, Xu Y (2018). Non-invasive monitoring of in vivo hydrogel degradation and cartilage regeneration by multiparametric MR imaging. Theranostics.

[26] Balakrishnan VS, Rao M, Kausz AT, Brenner L, Pereira BJ, Frigo TB (2009). Physicochemical properties of ferumoxytol, a new intravenous iron preparation. Eur J Clin Invest.

[27] Landry R, Jacobs PM, Davis R, Shenouda M, Bolton WK (2005). Pharmacokinetic study of ferumoxytol: a new iron replacement therapy in normal subjects and hemodialysis patients. Am J Nephrol.

[28] Lu M, Cohen MH, Rieves D, Pazdur R (2010). FDA report: Ferumoxytol for intravenous iron therapy in adult patients with chronic kidney disease. Am J Hematol.

[29] Neuwelt EA, Varallyay CG, Manninger S, Solymosi D, Haluska M, Hunt MA (2007). The potential of ferumoxytol nanoparticle magnetic resonance imaging, perfusion, and angiography in central nervous system malignancy: a pilot study. Neurosurgery.

[30] Pai AB, Nielsen JC, Kausz A, Miller P, Owen JS (2010). Plasma pharmacokinetics of two consecutive doses of ferumoxytol in healthy subjects. Clin Pharmacol Ther.

[31] Toth GB, Varallyay CG, Horvath A, Bashir MR, Choyke PL, Daldrup-Link HE (2017). Current and potential imaging applications of ferumoxytol for magnetic resonance imaging. Kidney Int.

[32] Corot C, Robert P, Idee JM, Port M (2006). Recent advances in iron oxide nanocrystal technology for medical imaging. Adv Drug Deliv Rev.

[33] Juweid ME, Cheson BD (2006). Positron-emission tomography and assessment of cancer therapy. N Engl J Med.

[34] Nejadnik H, Castillo R, Daldrup-Link HE (2013). Magnetic resonance imaging and tracking of stem cells. Methods Mol Biol.

[35] Dominici M, Le Blanc K, Mueller I, Slaper-Cortenbach I, Marini F, Krause D (2006). Minimal criteria for defining multipotent mesenchymal stromal cells. The International Society for Cellular Therapy position statement. Cytotherapy.

[36] Metz S, Bonaterra G, Rudelius M, Settles M, Rummeny EJ, Daldrup-Link HE (2004). Capacity of human monocytes to phagocytose approved iron oxide MR contrast agents in vitro. Eur Radiol.

[37] Heyn C, Bowen CV, Rutt BK, Foster PJ (2005). Detection threshold of single SPIO-labeled cells with FIESTA. Magn Reson Med.

[38] Henning TD, Sutton EJ, Kim A, Golovko D, Horvai A, Ackerman L (2009). The influence of ferucarbotran on the chondrogenesis of human mesenchymal stem cells. Contrast Media Mol Imaging.

[39] Daldrup-Link HE, Nejadnik H (2014). MR Imaging of Stem Cell Transplants in Arthritic Joints. J Stem Cell Res Ther.

[40] Liu A, Yu T, Young K, Stone N, Hanasoge S, Kirby TJ (2020). Cell Mechanical and Physiological Behavior in the Regime of Rapid Mechanical Compressions that Lead to Cell Volume Change. Small.

[41] Wolfs E, Struys T, Notelaers T, Roberts SJ, Sohni A, Bormans G (2013). 18F-FDG labeling of mesenchymal stem cells and multipotent adult progenitor cells for PET imaging: effects on ultrastructure and differentiation capacity. J Nucl Med.

[42] Kang WJ, Kang HJ, Kim HS, Chung JK, Lee MC, Lee DS (2006). Tissue distribution of 18F-FDG-labeled peripheral hematopoietic stem cells after intracoronary administration in patients with myocardial infarction. J Nucl Med.

[43] Liu A, Islam M, Stone N, Varadarajan V, Jeong J, Bowie S (2018). Microfluidic generation of transient cell volume exchange for convectively driven intracellular delivery of large macromolecules. Mater Today (Kidlington).

[44] Hallow DM, Seeger RA, Kamaev PP, Prado GR, LaPlaca MC, Prausnitz MR (2008). Shear-induced intracellular loading of cells with molecules by controlled microfluidics. Biotechnol Bioeng.

[45] Fechheimer M, Boylan JF, Parker S, Sisken JE, Patel GL, Zimmer SG (1987). Transfection of mammalian cells with plasmid DNA by scrape loading and sonication loading. Proc Natl Acad Sci U S A.

[46] Liu Y, Yan J, Prausnitz MR (2012). Can ultrasound enable efficient intracellular uptake of molecules? A retrospective literature review and analysis. Ultrasound Med Biol.

[47] Marmottant P, Hilgenfeldt S (2003). Controlled vesicle deformation and lysis by single oscillating bubbles. Nature.

[48] Ohl CD, Arora M, Ikink R, de Jong N, Versluis M, Delius M (2006). Sonoporation from jetting cavitation bubbles. Biophys J.

[49] Capecchi MR (1980). High efficiency transformation by direct microinjection of DNA into cultured mammalian cells. Cell.

[50] Matsumoto D, Yamagishi A, Saito M, Sathuluri RR, Silberberg YR, Iwata F (2016). Mechanoporation of living cells for delivery of macromolecules using nanoneedle array. J Biosci Bioeng.

[51] Shalek AK, Robinson JT, Karp ES, Lee JS, Ahn DR, Yoon MH (2010). Vertical silicon nanowires as a universal platform for delivering biomolecules into living cells. Proc Natl Acad Sci U S A.

[52] Neumann E, Schaefer-Ridder M, Wang Y, Hofschneider PH (1982). Gene transfer into mouse lyoma cells by electroporation in high electric fields. EMBO J.

[53] Ding X, Stewart M, Sharei A, Weaver JC, Langer RS, Jensen KF (2017). High-throughput Nuclear Delivery and Rapid Expression of DNA via Mechanical and Electrical Cell-Membrane Disruption. Nat Biomed Eng.

[54] Boukany PE, Morss A, Liao WC, Henslee B, Jung H, Zhang X (2011). Nanochannel electroporation delivers precise amounts of biomolecules into living cells. Nat Nanotechnol.

[55] Lu CW, Hung Y, Hsiao JK, Yao M, Chung TH, Lin YS (2007). Bifunctional magnetic silica nanoparticles for highly efficient human stem cell labeling. Nano Lett.

[56] Arbab AS, Yocum GT, Kalish H, Jordan EK, Anderson SA, Khakoo AY (2004). Efficient magnetic cell labeling with protamine sulfate complexed to ferumoxides for cellular MRI. Blood.

[57] Arbab AS, Yocum GT, Rad AM, Khakoo AY, Fellowes V, Read EJ (2005). Labeling of cells with ferumoxides-protamine sulfate complexes does not inhibit function or differentiation capacity of hematopoietic or mesenchymal stem cells. NMR Biomed.

