# Supplementary material for: Instant labeling of therapeutic cells for multimodality imaging
Source: Theranostics. 2020 May 15;10(13):6024–34. doi: 10.7150/thno.39554 (PMC7255004; doi:10.7150/thno.39554)
Supplement: Supplementary file 1 — Supplementary methods and figures. [file thnov10p6024s1.pdf]

Instant labeling of therapeutic cells for multimodality imaging

Hossein Nejadnik[1#], Kyung Oh Jung[2#], Ashok J. Theruvath[1,3#], Louise Kiru[1], Anna Liu[4], Wei Wu[1], Todd Sulchek[4], Guillem Pratx[2], Heike E. Daldrup-Link[1,5¥]

[1] Department of Radiology, Molecular Imaging Program at Stanford, Stanford University, CA, 94305, USA

[2] Department of Radiation Oncology, Stanford University, CA, 94305, USA

[3] Department of Diagnostic and Interventional Radiology, University Medical Center of the Johannes Gutenberg-University Mainz, 55131 Mainz, Germany

[4] Department of Biomedical Engineering, Georgia Institute of Technology, Atlanta, GA, 30332, USA

[5] Department of Pediatrics, Stanford University, CA, 94305, USA

[#] These authors contributed equally.

[¥] **Corresponding author address**: Heike E. Daldrup-Link, Molecular Imaging Program at Stanford, Stanford University, 725 Welch Road, Stanford, CA 94304, **Email:** heiked@stanford.edu

Table of Contents

1. Materials and Methods

1.1. Cell migration of dual-labeled ADSCs

1.2. Cell proliferation of dual-labeled ADSCs

1.3. Cell immunomodulation of dual-labeled ADSCs

1.4. Cell differentiation of dual-labeled ADSCs

1.5. Cell labeling with nanoparticles comprised of different physiochemical properties

2. Supporting Figures

Figure S1. TEM image of ferumoxytol uptake in ADSCs

Figure S2. Comparison of control and conventional co-incubation of ferumoxytol with ADSCs

Figure S3. Comparison of mechanoporation and conventional co-incubation of ^{18}F-FDG with ADSCs

Figure S4. Comparison of the cell migration ability of unlabeled and dual-labeled ADSCs

Figure S5. Comparison of the cell proliferation ability of unlabeled and dual-labeled ADSCs

Figure S6. Comparison of the immunomodulation ability of unlabeled and dual-labeled ADSCs

Figure S7. *In vitro* differentiation of ADSCs into chondrocytes.

Figure S8. Comparison of co-incubation and mechanoporation labeling of ADSCS with different nanoparticles.

1. Materials and Methods

1.1. Cell migration of dual-labeled ADSCs

Boyden Chamber assay was performed to investigate the effect that dual labeling has on the migration of ADSCs. Briefly, Polyethylene Terephthalate (PET) hanging cell culture inserts (Millipore, MCEP24H48) were placed in a 24-well plate and 100uL serum free medium were added to each well. After dual-labelling, cells ($2.5x10^5$ cells/mL) in serum free medium were aliquoted in each migration chamber. Culture medium supplemented with serum (750uL) was added to the lower chamber in the 24-well plate and the cells were incubated at 37ºC for 18 hours. Afterwards, the cells were fixed and stained with Shandon™ Kwik-Diff™ Stains kit (Fisher scientific, 9990701). Non-migrated cells were scrapped off with cotton swabs and migrated cells were imaged with a light microscope (Keyence, BZ-X710) and analyzed using image J.

1.2. Cell proliferation of dual-labeled ADSCs

Labeled and unlabeled ADSCs (2×10^4 cells/well) were seeded in Lab-Tek II Chamber Slide™ (ThermoFisher Scientific, USA). At day 5 after labeling, the cells were fixed by 4% formaldehyde and treated with the primary antibody overnight at 4°C as follows: anti-Ki67 antibody (1:100; ab15580, Abcam, Cambridge, UK). The secondary antibody was the anti-Rabbit IgG (H+L) Cross-Adsorbed Secondary Antibody, Alexa Fluor 594 (1:100; A-21442, ThermoFisher Scientific, Santa Clara USA). The cells were imaged by fluorescence microscopy (EVOS FL, ThermoFisher Scientific, Santa Clara, CA, USA).

1.3. Cell immunomodulation of dual-labeled ADSCs

Labeled and unlabeled ADSCs (5×10^4/mL) were seeded in 24 well plates. Following adhesion of the ADSCs, the medium was aspirated and 2×10^5/mL peripheral blood mononuclear cells (PBMCs) in medium with or without phytohemagglutinin (PHA) were added. On the third day, the cell proliferation was assessed using a colorimetric Cell Counting Kit-8 (CCK-8, Sigma-Aldrich, St Louis, MO, USA).

\

1.4. Cell differentiation of dual-labeled ADSCs

Chondrogenic differentiation was performed as previously described. Briefly, 3×10^5 cells unlabeled and dual-labeled ADSCs were pelleted in 0.5 ml of serum-free chondrogenic differentiation medium (Lonza, PT-3003) and 10 ng/ml TGF-β1 (R&D Systems, 101B1001CF). Cells were maintained as a pellet and the medium was changed every 2 days. At 3 weeks, the pellets were embedded in optimal cutting temperature (OCT) compound and 5-μm-thick tissue slices were obtained and placed on glass slides. The slides were fixed with 4% formaldehyde and Alcian blue staining was performed.

1.5. Cell labeling with nanoparticles comprised of different physiochemical properties

To assess whether the labeling efficiency of the mechanoporation procedure is affected by the use of iron oxide nanoparticles with different physiochemical properties, we compared labeling with ferumoxytol to ferucarbotran (VivoTrax™, Magnetic Insight Inc, Alameda, CA; mean hydrodynamic diameter of 62 nm, Zeta potential of ~ −27.5 mV)[2] and Molday ION Evergreen (BioPal Inc™, Worcester, MA; mean hydrodynamic diameter 35 nm, Zeta Potential ~ +31 mV). Briefly, ADSCs (3 million/mL) were resuspended in flow buffer containing 2mg/mL of either ferumoxytol, ferucarbotran and Molday ION Evergreen. The cell and nanoparticle mixture either underwent co-incubation for 10 min or the mechanoporation labeling procedure using the microfluid system for 10 min. Afterwards, the cells were washed twice with PBS and iron levels were tested with inductively coupled plasma - optical emission spectrometry (ICP-OES).

2. Supporting Figures

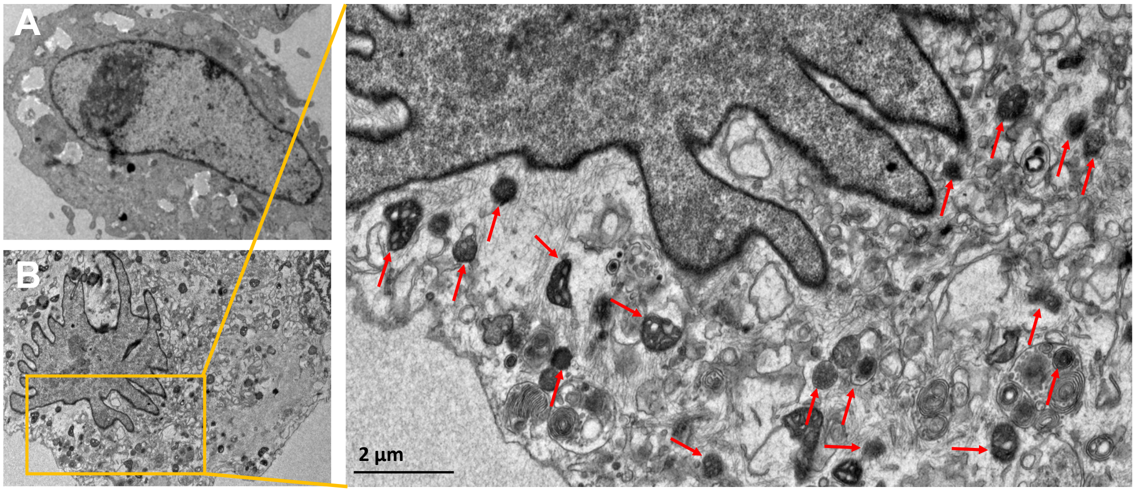

Figure S1. TEM image of ferumoxytol uptake in ADSCs. (A) Unlabeled ADSC shows no ferumoxytol uptake into the cytoplasm. (B) ADSC labeled with ferumoxytol by mechanoporation shows uptake of ferumoxytol nanoparticles into the cytoplasm (red arrows).

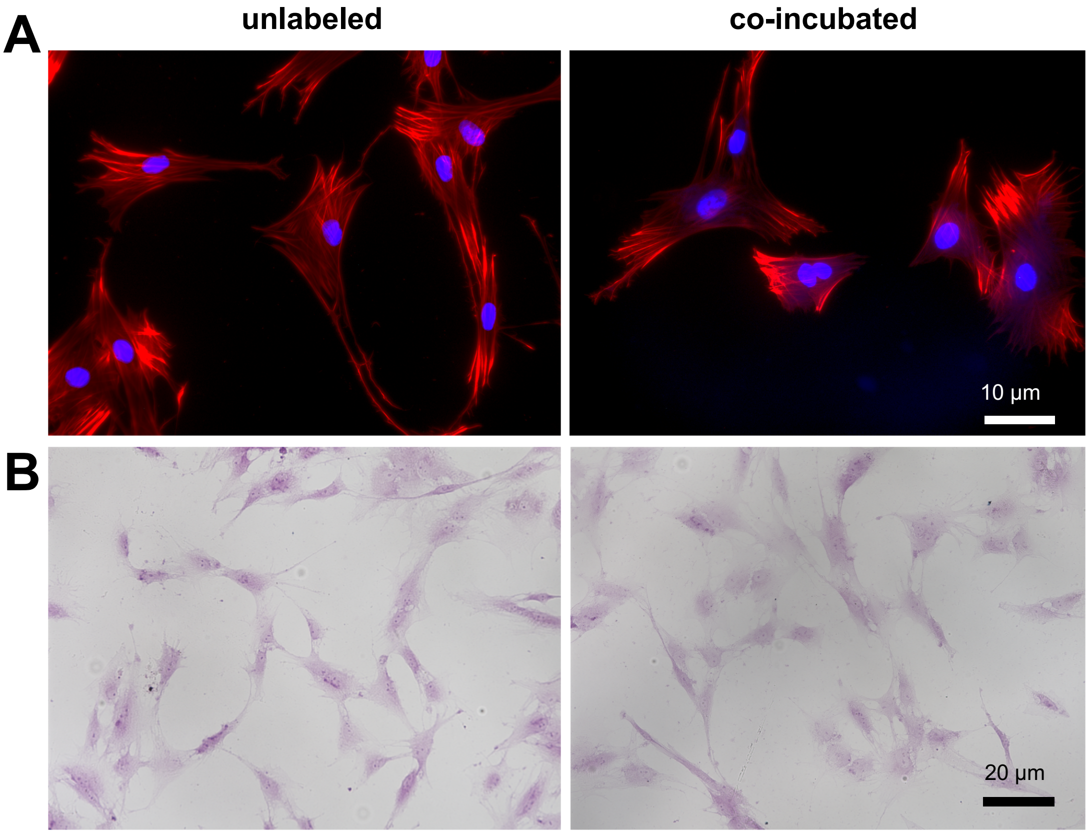

Figure S2. Comparison of control and conventional co-incubation of ferumoxytol with ADSCs. (A) Fluorescence microscopy of unlabeled and FITC-conjugated ferumoxytol co-incubated ADSCs show no iron uptake in both groups (blue represents nucleus, red represents cytoskeleton). (B) DAB-enhanced Prussian blue staining confirms absence of ferumoxytol uptake in both groups.

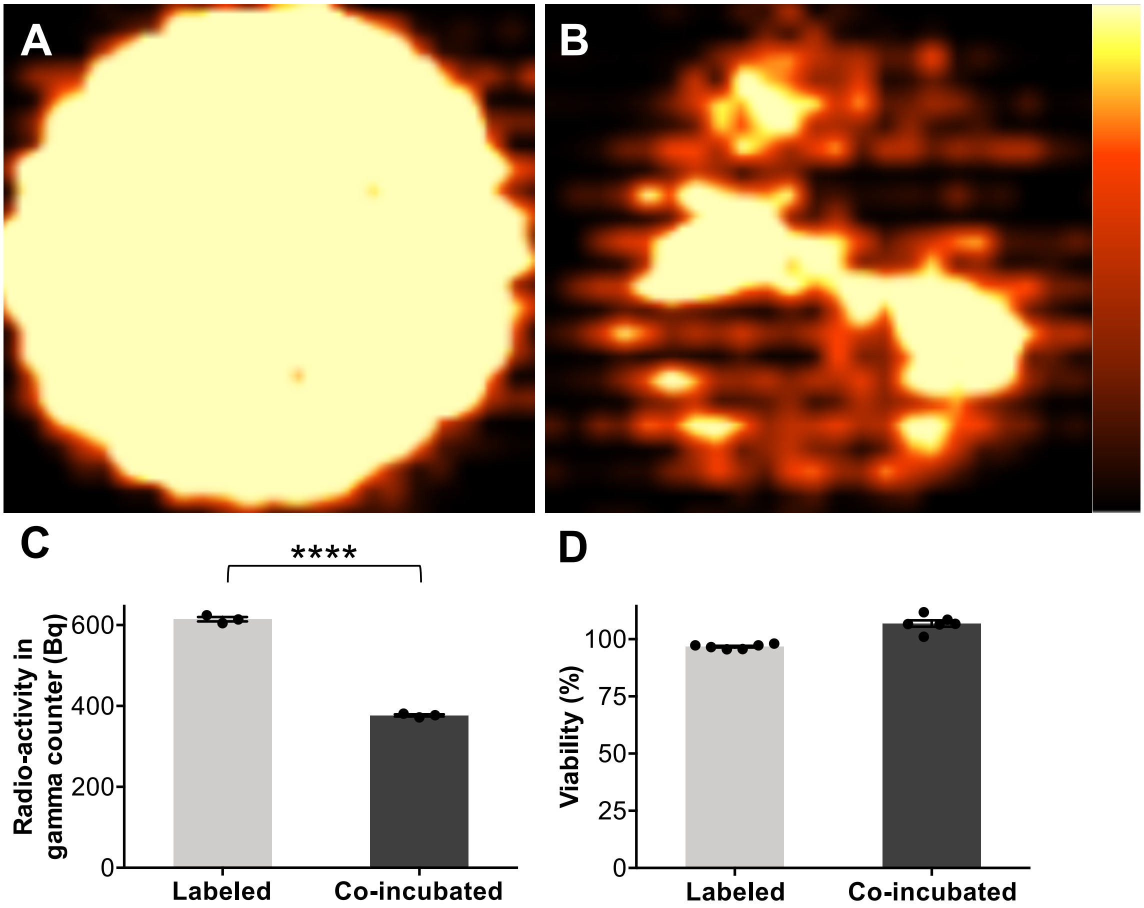

Figure S3. Comparison of mechanoporation and conventional co-incubation of ^{18}F-FDG with ADSCs. (A) ADSCs labeled with ^{18}F-FDG by mechanoporation show higher uptake in PET than (B) ADSCs labeled through conventional co-incubation. (C) Gamma counting confirmed the significantly higher ^{18}F-FDG labeling of ADSCs (614.3 ± 9.5 Bq / 1×10^4 cells) by mechanoporation compared with conventional co-incubation (376.6 ± 4.5 Bq / 1×10^4 cells) ($p < 0.0001$). (D) Colorimetric cell viability assay showed no significant difference between both methods. All data are means ± SEM; p values are from unpaired *t* test.

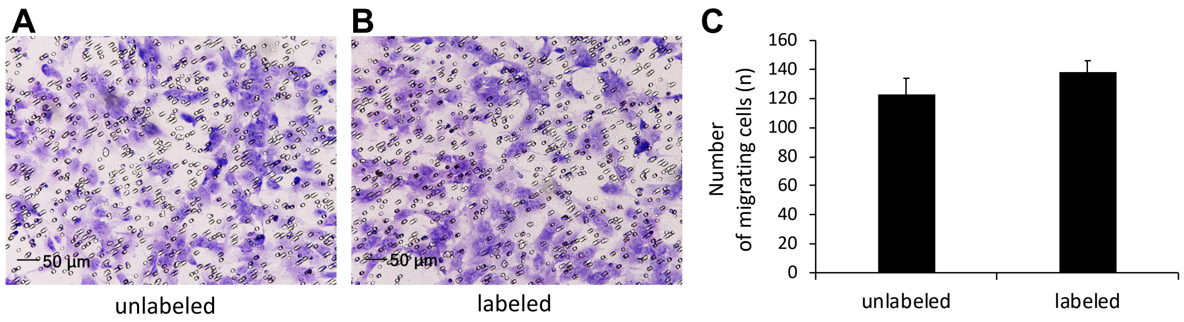

Figure S4. Comparison of the cell migration ability of unlabeled and dual-labeled ADSCs.

(A) Unlabeled and (B) dual-labeled ADSCs were stained for 18 hours after migration. No difference on migration was observed between dual-labeled and unlabeled cells. Magnification is 20X. (C) The quantification of migrated cells showed that there was no significant difference between dual-labeled and unlabeled cells.

.

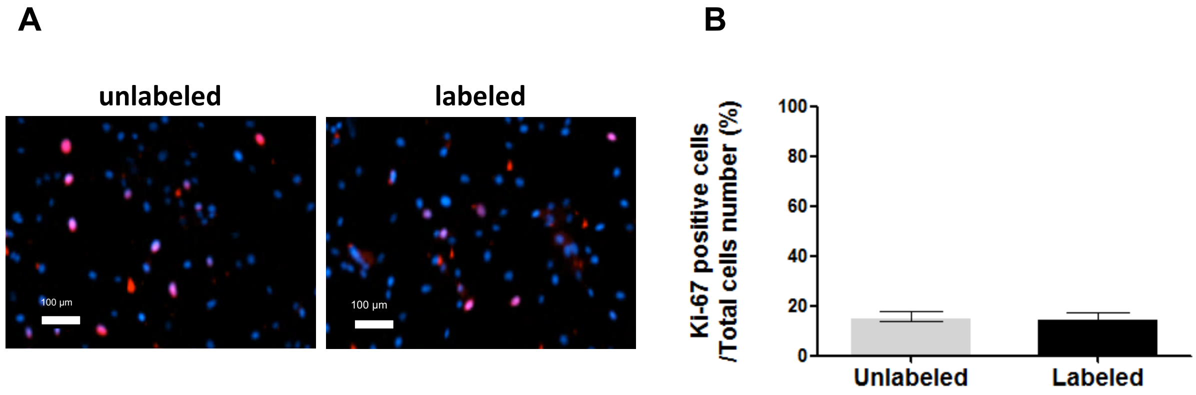

Figure S5. Comparison of the cell proliferation ability of unlabeled and dual-labeled ADSCs. (A) No difference on proliferation was observed between dual-labeled and unlabeled cells at 5 days after labeling. (B) The quantification of Ki-67 positive cells also showed that there was no significant difference between dual-labeled and unlabeled cells.

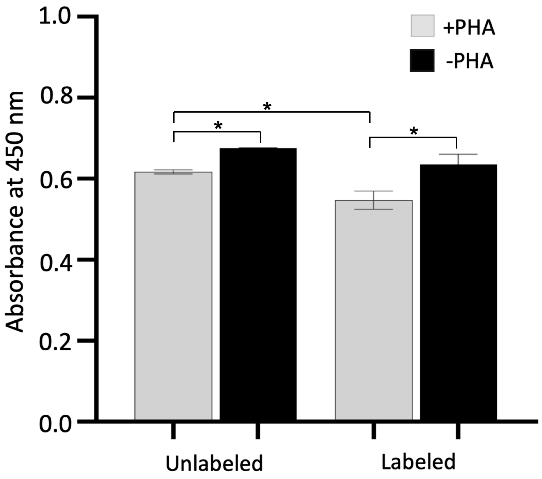

Figure S6. Comparison of the immunomodulation ability of unlabeled and dual-labeled ADSCs. Proliferation of PBMCs stimulated with PHA (unlabeled, 0.618 ± 0.004 and labeled, 0.548 ± 0.022) was significantly lower ($p < 0.05$) than PBMCs that were not stimulated with PHA (unlabeled, 0.675 ± 0.001 and labeled, 0.635 ± 0.015) in both co-cultures of dual-labeled and unlabeled ADSCs. The dual-labeled ADSCs retained their ability to suppress the proliferation of stimulated PBMCs demonstrated by the significantly lower ($p < 0.05$) proliferation of PBMCs stimulated with PHA and co-cultured with dual labeled ADSCs than PBMCs stimulated with PHA and co-cultured with unlabeled ADSCs.

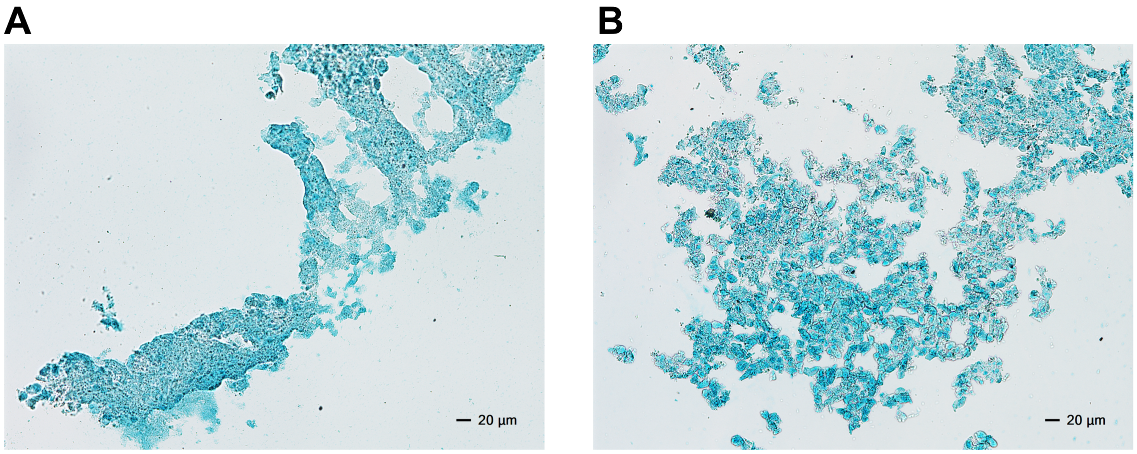

Figure S7. *In vitro* differentiation of ADSCs into chondrocytes. The pellets were kept in differentiation media for 3 weeks before embedded in OCT compound. Alcian blue staining revealed no difference in the differentiation of (A) unlabeled and (B) labeled ADSCs into chondrocytes.

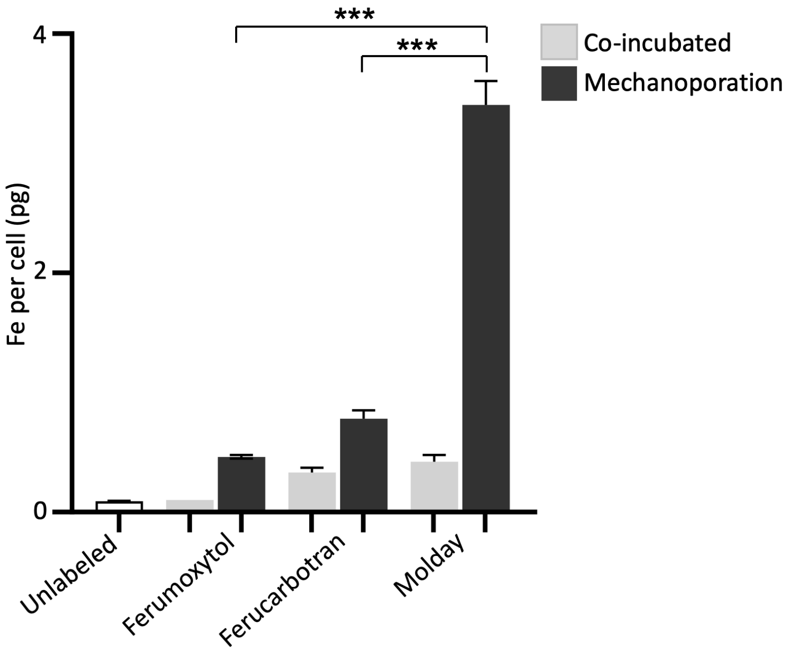

Figure S8. Comparison mechanoporation labeling of ADSCS with different nanoparticles.

ADSCs were labeled with iron oxide nanoparticles that have different physiochemical properties. Ferumoxytol, ferucarbotran and molday ION evergreen showed 0.1 ± 0.001 pg per cell, 0.33 ± 0.023 pg per

cell and 0.42 ± 0.033 pg per cell with the co-incubation method as determined by ICP-OES. The uptake of ferucarbotran (0.78 ± 0.040 pg per cell) following mechanoporation was two-fold greater than labeling with ferumoxytol (0.46 ± 0.015 pg per cell). Whilst the uptake of the molday ION evergreen (3.4 ± 0.200 pg per cell) was significantly greater ($p<0.0001$) than labeling with ferumoxytol and ferucarbotran. All data are means ± SEM; p values are from one-way analysis of variance.
